# Proximal tibia for alveolar augmentation and augmentative rhinoplasty—a suitable option? A retrospective clinical study on donor and recipient site morbidity

**DOI:** 10.1186/s13005-024-00470-w

**Published:** 2024-10-30

**Authors:** Paula Korn, Anastasia Melnikov, Matthias Kuhn, Samaneh Farahzadi, Günter Lauer, Tom Alexander Schröder

**Affiliations:** 1 Department of Oral and Maxillofacial Surgery, University Hospital and Faculty of Medicine Carl Gustav Carus, Technische Universität Dresden, Dresden, Germany; 2 Institute for Medical Informatics and Biometry, Faculty of Medicine Carl Gustav Carus, Technische Universität Dresden, Dresden, Germany

**Keywords:** Tibia bone graft, Alveolar ridge augmentation, Augmentative rhinoplasty, Doner site morbidity

## Abstract

**Background:**

Autologous bone grafts are essential in reconstructive oral and maxillofacial surgery, and depending on the donor site, they can be associated with specific harvesting morbidities. One of the most commonly applied bone grafts is the iliac crest bone graft, irrespective of other grafts, which might be associated with an easier surgical procedure or the possibility of harvesting them under local anaesthesia. Objective of the study is the clinical evaluation of proximal tibia bone grafts regarding their eligibility for maxillofacial bone grafting.

**Methods:**

In this retrospective study, proximal tibia bone grafts were examined with regard to associated donor and recipient site morbidity and their suitability for alveolar ridge augmentation and rhinoplasty.

**Results:**

In total, 21 tibia grafts were included. Fifty-seven percent of the bone grafts were used for alveolar ridge reconstruction, and 43% were used for augmentative rhinoplasty. No significant complications occurred during or after harvesting, but in 14.3% of the patients, minor wound healing disorders were recorded at the donor site, and in 19% of the patients, they were recorded at the recipient site. Statistically, patient sex, age, nicotine and alcohol abuse and metabolic diseases had no significant influence on the complication rate. Graft harvesting under local anaesthesia and at summer temperatures was associated with significantly more complications at the harvesting site (p < 0.05). In cases of dental implant insertion into augmented sites, the implants (*n* = 31) were followed up for a median period of 40.5 months, during this time 86.7% of the implants survived.

**Conclusion:**

The proximal tibia is a suitable donor site for harvesting autologous bone grafts for alveolar ridge augmentation or rhinoplasty because the donor site morbidity is low, and in contrast to iliac crest bone grafts, they can be harvested under local anaesthesia, which might be advantageous for outpatient surgeries.

## Introduction

Autologous bone grafts are elementary components of reconstructive oral and maxillofacial surgery due to their osteoconductive, osteoinductive and osteogenic characteristics [11, 17, 19, 21, 29]. Depending on the indication and required amount of tissue, different autologous bone grafts can be considered and they can be classified according to their morphology as cortical, cancellous, corticocancellous or vascularized bone grafts [31]. The established harvesting locations for non-vascularized grafts are the alveolar bone, iliac crest, proximal or distal part of the tibia, cranial bone and rib [9, 31]. Intraoral bone grafts are advantageous for alveolar reconstruction because the donor and recipient sites are structurally and spatially close. Nevertheless, the amount of harvestable bone is limited, and extraoral bone grafts might be indicated [3]. The indications for extraoral, non- vascularized bone grafts for alveolar reconstruction are large and complex intra- and extrabony alveolar bone defects and general alveolar atrophy due to multiple teeth loss combined with a non-availability of enough harvestable intraoral bone grafts or contraindications for intraoral bone harvesting, e.g. limited oral mucosa. The iliac crest represents the most common donor site for autologous bone grafts. Nevertheless, there is associated donor site morbidity [5, 20]. Possible minor and major complications include persistent donor-site pain, hematoma formation, incisional hernia, donor site fracture, nerve injury and infections [31]. The reported rates of complications after harvesting iliac crest bone grafts are 10% minor and 5.8% major complications on the basis of a review of 414 cases [2]. Younger and Capman published complication rates of 23.5% minor and 6.2% major complications [37].

Since 1914, the proximal tibia has been known as a possible donor site for corticocancellous bone grafts [36]. Comparisons of the tibia and iliac crest as donor regions can be found in the literature [6, 22]. The authors agreed that the tibia region offers clear advantages. The surgical approach can be small with reduced scaring afterwards, postoperative discomfort in the donor region is minimal, and the tibia can be loaded again after a few days [6, 22, 35]. Another advantage compared with iliac crest bone grafts is the option to perform surgery under local anaesthesia, which prevents patients from experiencing general anaesthesia-related risks and reduces the cost of surgery [3, 9]. This might be relevant in times of increased cost consciousness in the healthcare system. The osseous healing of the donor site after graft harvesting is also uneventful, and Vanryckeghem et al. reported the potential to form new cancellous bone after cancellous bone graft harvesting [33]. However, potential complications and donor site morbidities, can also be related to proximal tibia bone grafts. O´Keeffe et al. published one of the earliest retrospective studies on donor site morbidity and reported a complication rate of 1.3% after a follow-up period of 20.4 months for 230 harvesting procedures. This result is in line with the 1.9% rate of complications published by Alt et al. after a follow-up of 26.4 weeks [1] and the 1.6% reported by Frohberg and Mazock [13]. Interestingly, most patients underwent bone grafting for orthopedic indications, and less data are available for maxillofacial augmentation procedures; moreover, complications at the recipient site are rarely reported. Owing to the anatomical structure of the tibia, a cancellous bone graft can be harvested for jaw augmentation, as can a cortical graft for augmentative rhinoplasty [14]. Depending on the required graft, numerous harvesting techniques are established, for example, cancellous bone can be excavated via a trepan drill, or cortical chips can be prepared via a bone saw. This leads to the question of whether there is a statistical context between donor site morbidity and indications for bone augmentation and other patient-related factors.

### Aim

The aim of this study was to evaluate complications at the donor and recipient sites after harvesting tibia bone grafts for alveolar augmentation and augmentative rhinoplasty. In addition, possible patient- and methodical-related factors were analysed for their influence on the complication rate. In patients with dental implant insertion, implant survival was monitored.

## Methods

This retrospective study analysed patients treated with an autologous tibia bone graft in the clinic for Oral and Maxillofacial Surgery at University Hospital Carl Gustav Carus Dresden, Germany, between 2004 and 2017. The study was approved by the ethics committee (BO-EK-374082020) and followed the World Medical Association Declaration of Helsinki (1964), which was last updated in 2013.

### Patient inclusion and exclusion criteria

The inclusion criteria were adult and consenting patients, medical indications and informed consent for alveolar bone augmentation using an autologous bone graft from the proximal tibia or medical indications, and informed consent for augmentative rhinoplasty in terms of nasal dorsum augmentation. The planning of the surgery, surgical realization, postoperative care and clinical follow-up were all performed at the same clinic.

The exclusion criteria were patients who were unable to consent independently and those who missed the clinical follow-up.

### Tibia bone graft harvesting technique

Bone was harvested from two sides of the tibial bone. Cancellous bone is usually obtained from the tibia head in an outpatient setting under local anaesthesia (lidocaine 2%) without sedation. The cortical bone chip was taken from the anterior border of the upper proximal tibia, and concomitant rhinoplasty was performed under general anaesthesia.

**Fig. 1:**
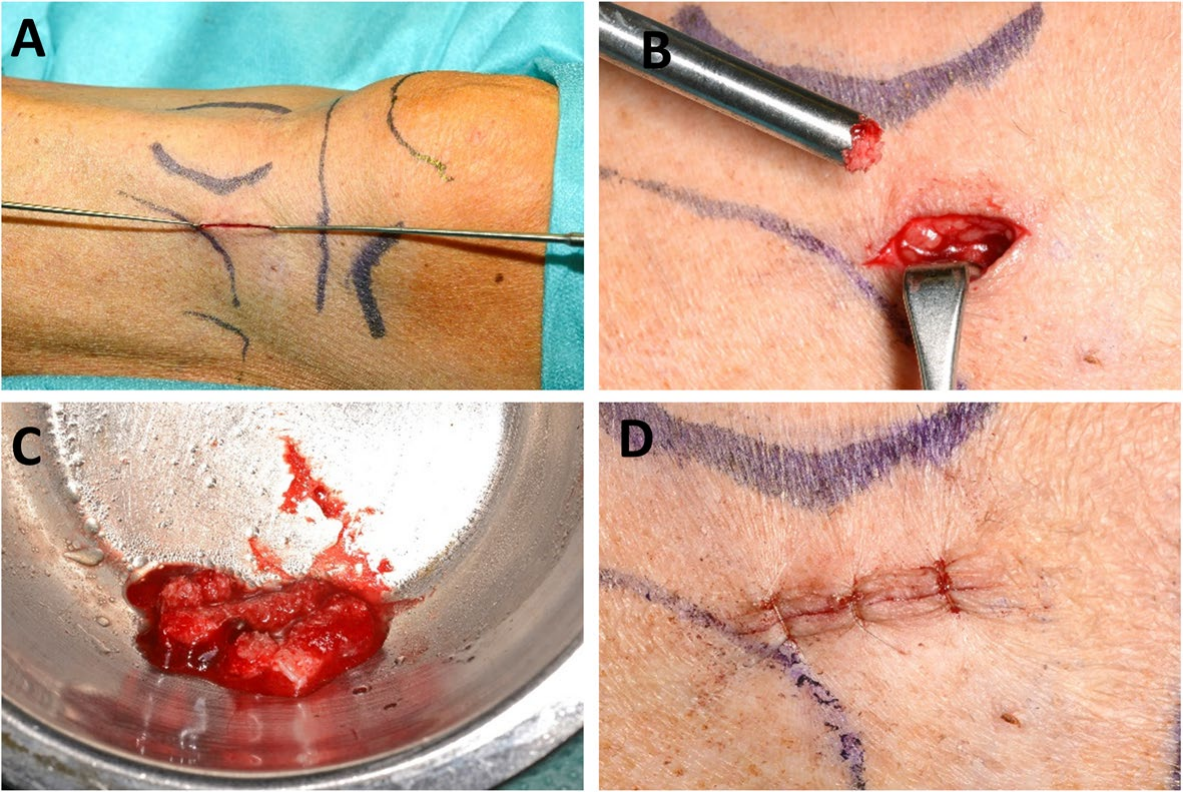
A corticocancellous cylinder bone graft from the anterior tibia was harvested. After a cutaneous incision (**A**), a corticocancellous graft can be harvested using a trepan drill (**B**). The amount of bone graft can be adapted to the planned augmentation (**C**). Suturing of the wound (**D**)

Cancellous bone cylinders were harvested from the lateral tibia condyle via an access lying roughly at the height of the tibial tuberosity via a hand trephine drill (Aesculap, Tuttlingen, Germany) (Fig. 1). After a 1 cm long parasagittal incision, subcutaneous dissection and splitting of the periosteum at the upper insertion of the tibialis anterior muscle were performed, and the bone surface was exposed. Using a hand trephine burr, the cortical bone was cut, and the first cortico-cancellous bone cylinder was secured (Fig. 1 B). More cancellous bone cylinders were taken while advancing the burr in different horizontal directions within the tibia head. In addition, the spongiosa was taken from the inside of the tibial head through this round bony window with bone curettes (Fig. 1 C). The wound was closed layer by layer, and the skin was sutured interrupted using resorbable Monocryl® (Ethicon, Johnson & Johnson Medical GmbH, Norderstedt, Germany) (Fig. 1 D).

To harvest the cortical bone chip, a parasagittal 6 cm long skin incision lateral to the anterior tibial border was made 4 cm below the tibial tuberosity. After tissue dissection, the tibia bone 5 mm lateral–posterior to the border was reached. Here, the periosteum was opened parallel to the anterior border, and a 5 cm long bone chip was cut using a jigsaw or an ultrasonic jaw. Then, a 5 cm long cortical bone chip with a triangular shape in the cross section of the anterior tibia border was lifted off. This approach enables the periosteum and connective tissue to be left attached to the anterior tibial border bone to upholster the nasal bridge. Prior to layered wound closure, the pointy tibia bone edges were smoothed with a 3 mm diamond burr.

### Analysed data

A medical history was obtained from all patients, and a clinical examination was performed. The collected data were entered into the documentation programs of the clinic and outpatient departments: Orbis (DH Healthcare GmbH, Version 03.15.02.00, 53,227 Bonn, Germany), Dentware (Dentware Computer GmbH, Version 1.3n (19.01.2024), 82,216 Maisach, Germany) and Sidexis (Sidexis XG 2.63, Dentsply Sirona Deutschland GmbH, Bensheim, Germany).

In addition to the age and sex of the patient, the patient’s medical history included general health status in terms of hypertonia, diabetes mellitus, hyperlipidaemia, and thyroid disorders. A standardized medical history questionnaire to be completed by the patients themselves was used to obtain the patients’ general medical history. The patients were also asked about their smoking behavior at the time of the surgery without evaluating their pack-years. The same was done for alcohol consumption. Both parameters were included in the statistical analysis as yes/no decisions due to the small sample size.

During the treatment of patients, possible complications before, within or after surgery were reported in a particular patient file. The complications were divided into major and minor complications. The major complication is bone fracture at the donor site, a life-threatening condition that requires hospitalization or the loss of the bone graft after transplantation. Minor complications included all abnormalities that required outpatient medical and/or drug intervention and/or persisted for more than two weeks, e.g., local wound infections.

In cases with dental implant insertion the implant type, implant position and time point of implant insertion after augmentation were documented. Clinical implant success criteria are: implant is in situ and prosthodontically loaded. Besides that, the panoramic or dental X-ray shows a sufficient osseointegration of at least 75% of the implant length.

### Statistics

All the data were analysed with Microsoft Excel (version 2016; Microsoft Corporation, Redmond, WA, USA) and R (version 4.3.1; R Foundation, Vienna, Austria). Descriptive statistics included the absolute and relative frequencies and the means and medians. We explored the associations between potential risk factors and the occurrence of complications in the donor, recipient and jaw regions after implant placement. To this end, we applied Firth’s bias-reduced logistic regression to estimate odds ratios (ORs) with Haldane correction. This method was specifically developed for small samples and yields meaningful ORs in the presence of zero count cells [16]. Confidence intervals for the OR and associated P values were derived from Firth's logistic regression using profile-penalized likelihood. The null hypothesis was that there is no association between the two parameters. The level of statistical significance was set at alpha = 0.05. Implant survival was calculated via Kaplan‒Meier analysis, and the reverse Kaplan‒Meier method was applied to estimate the median follow-up of the dental implants at years 1, 3, 5 and 10 after insertion.

## Results

### Age, sex and medical history

In this study, 19 patients were included. Two of the 19 patients received a second tibia bone graft independently from the first surgery; thus, 21 donor sites and 21 recipient sites were included. The average age of the patients was 49.9 years (from 17 to 69 years). The sex distribution was almost equal (10 male patients and 9 female patients; one male patient and one female patient received 2 grafts each). We observed no statistically significant associations between sex and surgical complications in the donor (OR 0.6, *p* = 0.65), recipient (OR 1.12, *p* = 0.91) or implant site (OR 1.3, *p* = 0.84) (Tabs. 1, 2, 3). To test for the influence of age on the complication rate, the patients were divided into two groups (> 45 years, < 45 years), and there were strong effects, albeit not statistically significant (donor site OR 2.85, *p* = 0.47; recipient site OR 0.24, *p* = 0.19; alveolar bone after dental implant insertion OR 1.57, *p* = 0.74).

All patients were asked about their current smoking behavior at the time of surgery; 57.1% of the patients did not smoke, and 42.9% reported nicotine consumption, which was 9.5% 1–4 cigarettes/day, 28.6% 5–20 cigarettes/day, and 4.8% more than 20 cigarettes/day. However, patients with nicotine abuse had no significantly higher complication rates than those without nicotine abuse at both the donor site (OR 2.56, *p* = 0.4) and the recipient site (OR 1.4, *p* = 0.74) (Tables 1, 2).

**Table 1:** Overview of patient- and surgery-related factors with regard to their impact on the complication rate at the tibia donor site

**Predictor**	**N**	**Proportion Complication**	**OR**	**P-value**	**CI**
Sex (female/male)	21 (f = 10 vs m = 11)	f = 10% vs m = 18.2%	0.6	0.6474	0.048 — 5.411
Age (> 45 years, < 45 years)	21 (> 45 years = 16 vs < 45 years = 5)	> 45 = 18.8% vs < 45 = 0%	2.852	0.469	0.217 — 408.107
Smoking (yes/no)	21 (yes = 9 vs no = 12)	yes = 22.2% vs no = 8.3%	2.556	0.3999	0.282 — 32.25
Alcohol consumption (yes/no)	17 (yes = 8 vs no = 9)	yes = 25% vs no = 11.1%	2.179	0.4941	0.231 — 28.413
Comorbidity (yes/no)	21 (yes = 10 vs no = 11)	yes = 0% vs no = 27.3%	0.1156	0.1004	0.001 — 1.441
Season (> 20°C, < 20°C)	21 (> 20°C = 8 vs < 20°C = 13)	> 20°C = 37.5% vs < 20°C = 0%	17.18	**0.02668**	1.342 — 2464.329
Anaesthesia form (local vs. general)	21 (local = 4 vs general = 17)	local = 50% vs general = 5.9%	11	**0.0456**	1.049 — 169.36

*OR* odds ratio, *CI* confidence interval

**Table 2:** Overview of patient- and surgery-related factors with regard to their impact on the complication rate at the recipient site

**Predictor**	**N**	**Proportion Complication**	**OR**	**P-value**	**CI**
Sex (female/male)	21 (f = 10 vs m = 11)	f = 20% vs m = 18.2%	1.118	0.9124	0.142 — 8.833
Age (> 45 years, < 45 years)	21 (> 45 years = 16 vs < 45 years = 5)	> 45 = 12.5% vs < 45 = 40%	0.2414	0.1875	0.026 — 2.066
Smoking (yes/no)	21 (yes = 9 vs no = 12)	yes = 22.2% vs no = 16.7%	1.4	0.7402	0.177 — 11.153
Alcohol consumption (yes/no)	17 (yes = 8 vs no = 9)	yes = 12.5% vs no = 33.3%	0.3714	0.3589	0.03 — 2.997
Comorbidity (yes/no)	21 (yes = 10 vs no = 11)	yes = 20% vs no = 18.2%	1.118	0.9124	0.142 — 8.833
Season (> 20°C, < 20°C)	21 (> 20°C = 8 vs < 20°C = 13)	> 20°C = 12.5% vs < 20°C = 23.1%	0.6	0.6319	0.05 — 4.616
Anaesthesia form (local vs. general)	21 (local = 4 vs general = 17)	local = 25% vs general = 17.6%	1.776	0.6223	0.14 — 16.094

*OR* odds ratio, *CI* confidence interval

The question about alcohol consumption was a yes/no decision, and 47.6% reported alcohol consumption, whereas 33.3% did not consume alcohol. Nineteen percent of patients made no statement about alcohol consumption. There was no significant association between alcohol consumption and complications at the donor site (*p* = 0.49), recipient site (*p* = 0.36) or dental implant site (*p* = 1). Nevertheless, the rate of complications was slightly increased at the donor site (OR 2.18) and implant site (OR 1.67) for patients who consumed alcohol. At the recipient site, the OR was 0.37 (Tables 1, 2 and 3).

**Table 3:** Overview of patient- and surgery-related factors with regard to their impact on the complication rate at the dental implant site after augmentation using tibia bone grafts

**Predictor**	**N**	**Proportion Complication**	**OR**	**P-value**	**CI**
Sex (female/male)	10 (f = 3 vs m = 7)	f = 66.7% vs m = 57.1%	0.8355	0.8355	0.111 — 19.438
Age (> 45 years, < 45 years)	10 (> 45 = 8 vs < 45 = 2)	> 45 = 62.5% vs < 45 = 50%	0.7364	0.7364	0.096 — 26.31
Smoking (yes/no)	10 (yes = 5 vs no = 5)	yes = 60% vs no = 60%	1	>0.999	0.094 — 10.645
Alcohol consumption (yes/no)	9 (yes = 4 vs no = 5)	yes = 75% vs no = 60%	1.667	0.6877	0.137 — 26.249
Comorbidity (yes/no)	10 (yes = 4 vs no = 6)	yes = 50% vs no = 66.7%	0.5556	0.6209	0.047 — 5.905
Season (> 20°C, < 20°C)	10 (> 20 °C = 3 vs < 20 °C = 7)	> 20 °C = 66.7% vs < 20 °C = 57.1%	1.296	0.8355	0.111 — 19.438

All dental implants were inserted in local anaesthesia

*OR* odds ratio, *CI* confidence interval

Regarding their medical history, 47.6% of patients reported a medical condition associated with daily medication (60% female, 40% male patients). The conditions were hypertonia (19%), hyperlipidaemia (19%), thyroid disorders (19%) and diabetes mellitus (4.8%). There was no statistically significant correlation between a general disease requiring medication and a possible complication in the current study. Nevertheless, the OR for complications for patients with comorbidities was greater at the recipient site than at the donor site (donor site OR 0.12, *p* = 0.10) or recipient site (OR 1.12, *p* = 0.91) (Tables 1 and 2).

### Indications for bone grafting and surgery-related considerations

**Fig. 2:**
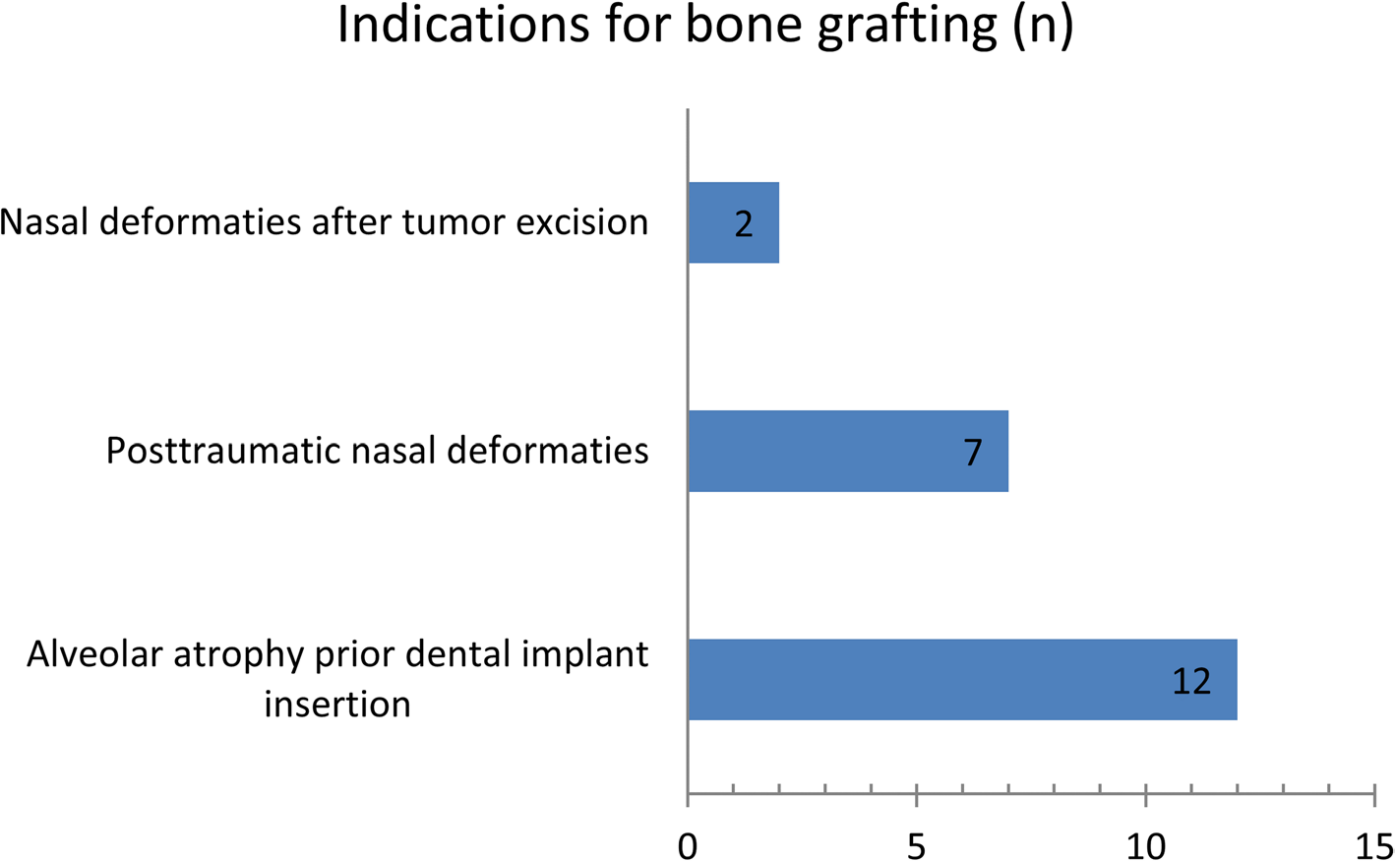
Indications for grafting surgeries using tibia bone grafts

In total, 21 tibia bone grafts were applied, and the left tibia was chosen in 76% and the right tibia in 24% of the patients as the donor site. In 12 of the patients, the indication was atrophic alveolar bone with planned dental implant insertion for oral rehabilitation. An augmentative rhinoplasty was necessary in 9 patients, and the indications were posttraumatic deformations of the nose or nasal deformations after tumor excision (Fig. 2).

The bone graft harvesting procedures from the proximal tibia were performed mainly under general anaesthesia (17/21 cases, 81%), but 4 patients (19%) also underwent surgery under local anaesthesia. Compared with that of general anaesthesia, the complication rate at the donor site was 11-fold increased for local anaesthesia harvesting procedures compared to general anaesthesia, which was statistically significant (*p* = 0.05). The complication rate at the recipient site was not affected by the harvesting procedure (OR 1.78, *p* = 0.62). (Table 1).

Depending on the clinical indications, different types of tibia bone grafts can be harvested. In total, 21 tibia sites were used to harvest bone grafts for 21 recipient sites. For alveolar augmentation, in 50% of the cases, spongiosa cylinders or, in 16% of the cases, free spongious bone grafts were applied. For augmentative rhinoplasty, cortical tibia spans were chosen (34% of the cases), and the spans had lengths between 2.5 and 5 cm.

To identify possible relationships between the complications that occurred and the surgical conditions, the time of year was also considered. Sixty-two percent of the surgeries were performed during the cold season with outside temperatures below 20 °C, and 38% were performed during the summer season with temperatures above 20 °C. The complication rate significantly increased at the donor site when surgery was performed during the summer (OR 17.18, *p* = 0.03) (Table 1). The complication rate in the recipient area was not significantly affected (OR 0.6, *p* = 0.63) (Table 2).

### Complications

**Fig. 3:**
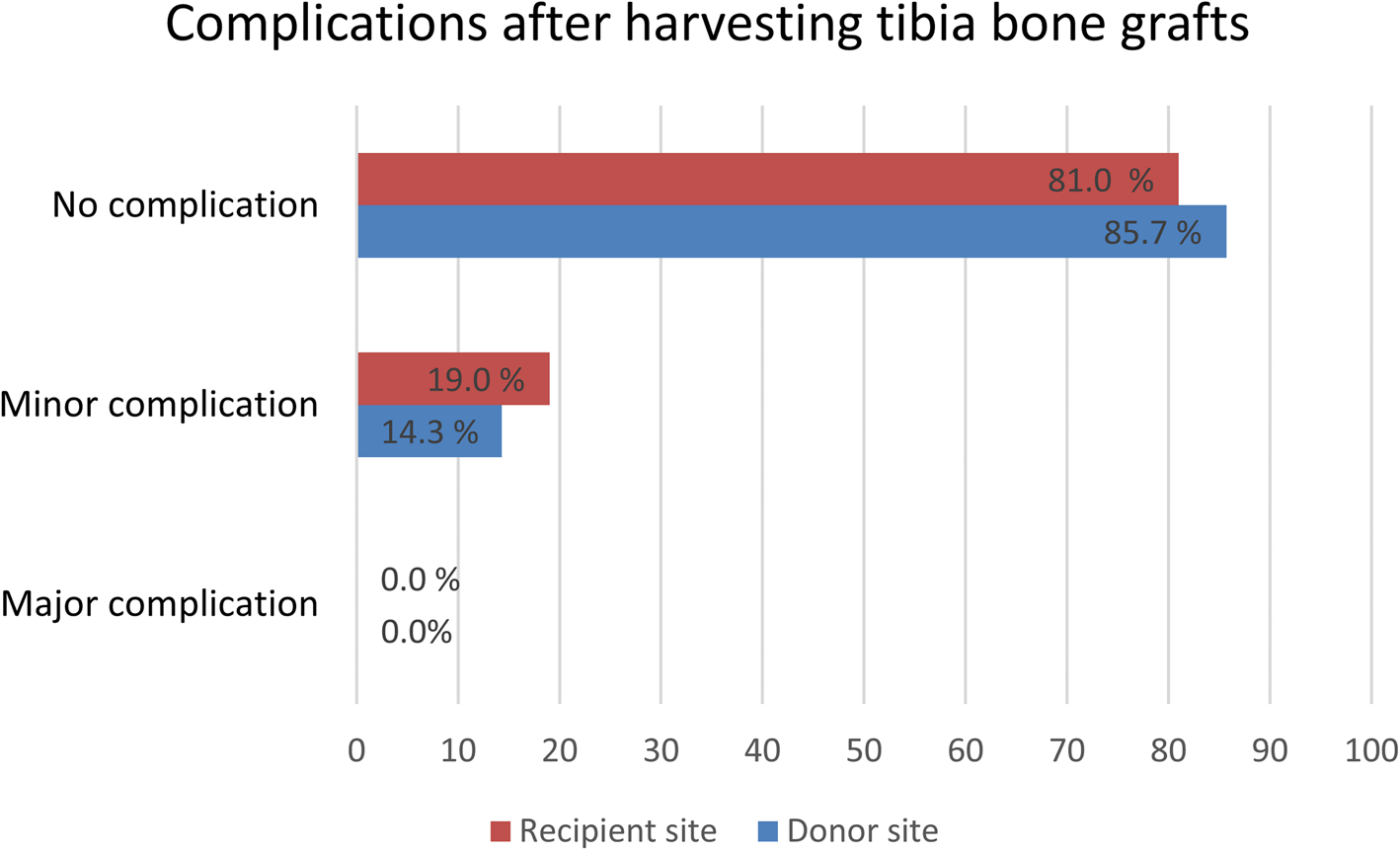
Percentage of complications after proximal tibia bone grafting (n_Donor site_ = 21, n_Recipient site_ = 21). No statistically significant difference was detected between the sites according to the McNemar test

Irrespective of the indication and bone graft type, no major complications occurred (Fig. 3). Minor complications were detected at both the donor and recipient sites. In 3 of the 21 cases, donor site complications occurred (14.3%): 2 patients (in the local anaesthesia group) developed local hyperthermia and pain, which was treated with drainage and antibiotics. One patient (in the general anaesthesia group) experienced prolonged wound healing with persistent edema for 3 months.

The minor complication rate at the recipient site was 18.1% because 4 alveolar sites had minor complications. Two patients presented with alveolar pain and swelling and required local antiseptic treatment and oral antibiotics. One patient had a wound dehiscence region 13–14, and one patient was temporarily hyposensitive to N. alveolaris inferior. All of them could be successfully treated, and no loss of the bone graft occurred. The mean duration of postsurgical surveillance was 27.6 months for all patients.

After augmentative rhinoplasty, 7 of 9 patients achieved good and satisfying clinical outcomes. In one patient, the cortical tibia span was slightly movable, but no further intervention was necessary. One graft showed a small lateral dislocation, and the direction of the tibia span was corrected during further, independently indicated, reconstructive surgery.

Statistically, no associations between the occurrence of a complication and the respective recipient regions were found. Nevertheless, the absolute number of minor complications at the recipient site was higher for alveolar augmentation than for rhinoplasty.

### Dental implant insertion into tibia bone grafts used for alveolar augmentation

In 9 patients, 31 dental implants were inserted after a mean period of 3.8 months after bone grafting. The following dental implant types were inserted: 65.5% Straumann (Straumann GmbH, Freiburg, Germany), 17.2% Xive (Dentsply Sirona Deutschland GmbH, Bensheim, Germany), 13.8% Camlog (Camlog Vertriebs GmbH, Wimsheim, Germany) and 3.5% Bicon (Bicon LLC, Boston, USA).

**Fig. 4:**
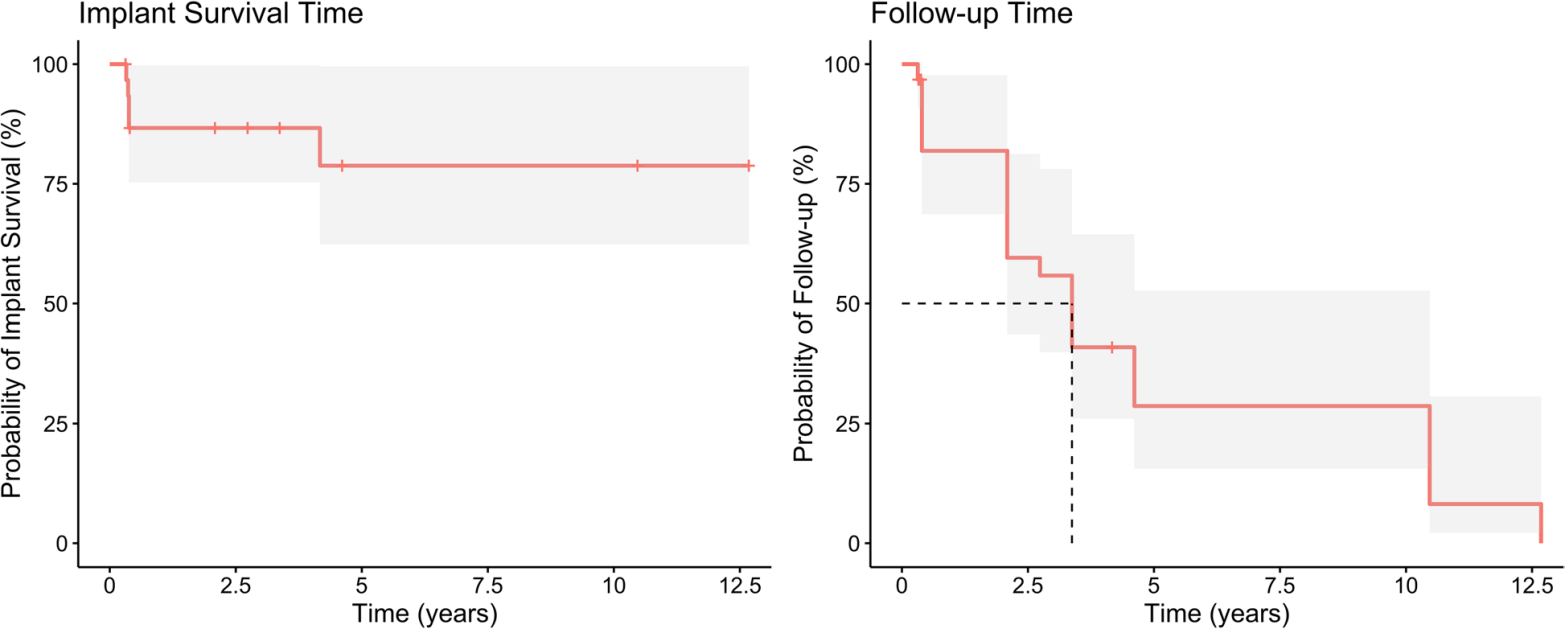
Dental implant survival time according to Kaplan‒Meier curve and follow-up time

The median follow-up after implant insertion was 40.5 months according to the reverse Kaplan‒Meier method, and within this period, 5 of the 31 implants showed no osseointegration and were removed (16.1%) (Table 4 and Fig. 4). The explantation of the 4 non-osseointegrated implants took place within the first 6 months after insertion, and the patients subsequently received new implants. In one case, the patient presented 4 years after insertion without the previously inserted implant, with no exact documentation of the time point of implant loss. The implant was not replaced. The rates of implant survival according to Kaplan‒Meier analysis after years 1, 3, 5 and 10 ranged from 86.7% after one year to 78.8% after 10 years (Table 4).

**Table 4:** Dental implant survival estimation. CI confidence interval

	Kaplan-Meier implant survival estimation				
Year	Implant count risk	Event	Censor	Survival	95%-CI
1	22	4	5	86.67%	75.32%–99.73%
3	15	0	7	86.67%	75.32%–99.73%
5	7	1	7	78.79%	62.37%–99.53%
10	7	0	0	78.79%	62.37%–99.53%

*CI* confidence interval

## Discussion

Donor site morbidity is a relevant medical issue if tissue needs to be harvested for reconstructive purposes. In general, it is defined as “the presence of persistent symptoms (≥ 1 year) after graft harvesting, as well as the need for subsequent intervention to treat complications related to the donor site.”[4].

### Complications at the donor site

The current retrospective study analysed the complication rate after harvesting tibia bone grafts for alveolar augmentation and augmentative rhinoplasty at the University Hospital Dresden, Germany, Department of Oral and Maxillofacial Surgery, within a mean follow-up period of 27.6 months. The first indication was for 57% of the patients, which was slightly more common than the second. Overall, no major complications occurred at the donor or recipient site. This finding is in line with the results of Kirmeier et al. [22] and Alt et al. [1], who also found no major donor site complications. Hughes et al. reported 2.7% major complications in terms of bone fractures [17]. Some minor complications at the donor site occurred in all studies, and the rate was 14.3% for alveolar augmentation and rhinoplasty in the present study, which is comparable to the 14.6% reported by Hughes et al. after 75 cases of tibia bone grafting for alveolar cleft augmentation were evaluated. A retrospective study including 8 patients published a rate of 12.5% [24]. A previous study with 230 cases, which represents the largest published cohort, reported a minor complication rate of 1.3% [32], whereas Frohberg and Mazock [13] have seen complications (mainly prolonged pain at the donor site and gait disturbance) in 19% of the 63 patients included in their retrospective study. Direct comparison of complication rates is difficult because several factors, such as clinical assessment of the wound, are subjectively influenced by the examiner, and standardized assessment is difficult. Furthermore, pain and discomfort are parameters with interindividual subjectivity. It seems that the complication rate decreases with increasing patient number in one study center, which can be explained by the increased experience of the surgeons and/or a more timesaving surgery. In our clinic, the average cut-seam time for harvesting a tibia bone graft was 31 min, which is comparable to the time reported by other groups [24, 27]. We found a significant correlation between donor site morbidity and climate. If the outside temperature was above 20°C, the minor complication rate significantly increased, and local wound infections occurred more frequently, which can be explained by the moist and warm wound area and, accordingly, increased microbial growth. Nonetheless, all of the minor complications of our study could be successfully treated, and no bone graft was lost during the follow-up period of 27.6 months.

### Complications at the recipient site

When the complications of the recipient site were assessed, differences between the 2 indications were detected. In total, 4 complications occurred, which represents 18.2% in total, 3 occurred after alveolar augmentation and just one after augmentative rhinoplasty. The alveolar indication has the challenge of microbial colonization of the oral cavity, which might have influenced the results, and it is questionable whether the type of bone graft caused the postsurgical hyposensitivity of N. mentalis. Excluding this case from the calculation, the rate was 2 of 12 cases (16.6%), which is the same result as that published by Atil et al., with the difference that they reported partial loss of the graft after alveolar augmentation [3]. In general, comparing studies evaluating alveolar augmentations before dental implant insertion is challenging because many surgical techniques and additional procedures, such as the use of membranes, have been established.

Considering the augmentative rhinoplasty separately, one case of delayed wound healing at the recipient site was detected, with no impact on graft survival. Owing to the small number of cases, 1 out of 10 represents a minor complication rate of 10% at the recipient site. Chauhan et al. reported favourable findings without any postoperative complications in a cohort of 9 patients [8], similar to the results published by Garcia‒Diez for 13 patients [14].

### Patient-related factors for complications

The success of a bone graft depends on various factors, and several factors were analysed in the current study regarding their impact on the complication rate. In summary, patient age, sex, smoking status and alcohol consumption were not significantly correlated with the complication rates at the donor and recipient sites. However, smoking clearly increased the risk of complications at the donor site 3.1-fold and 1.4-fold at the recipient site. A possible reason for the worsening of intraoral wound healing might be the reduced local blood microcirculation [15, 25]. The increased risk of, e.g., dental implant loss or marginal bone loss is well documented and confirmed in several reviews [12, 30]. This finding highlights the importance of preoperative awareness regarding the increased complication rate due to smoking. Frequent alcohol consumption is another factor associated with an increased risk of general postoperative morbidity, general infections, wound complications, pulmonary complications, prolonged stay at the hospital, and admission to the intensive care unit [10]. We found a 2.6-fold increased risk for complications at the donor or recipient site for patients who smoke. However, medical anamnesis regarding cardiovascular diseases or diabetes had no impact on the complication rate in the current study. The explanation might be that the patients were all under treatment for their general disease. In particular, poorly controlled diabetes is known for delayed wound healing of soft and hard tissues [23].

### Anaesthesia

Tibia bone grafts have the advantage of being harvestable under local anaesthesia [24] [22], but surgery is often performed under general anaesthesia. Dental implantology, in contrast to augmentative rhinoplasty, is usually performed under local anaesthesia; therefore, it would be advantageous if bone graft harvesting could also be performed under local anaesthesia. In our study, 4 of the 19 patients underwent surgery under local anaesthesia, and 2 minor complications in terms of delayed wound healing occurred at the donor site, while one occurred at the recipient site. Compared with those in the general anaesthesia group, more minor complications occurred at the donor site, but this difference was not statistically significant. This is in contrast to the literature, where tibia harvesting surgery under local anaesthesia was associated with 80% uneventful healing at the donor site, and only 5% of the 79 patients experienced complaints lasting longer than 2 weeks post-surgery [22].

### Dental Implant survival

The implant survival rate was 86,7% after a median follow-up period of 40.5 months post-implantation, which represents a slightly lower rate than that reported in other studies evaluating dental implant insertion into autologous bone grafts [7, 26, 28]. However, it seems to be relevant if intraoral local bone grafts or iliac crest bone grafts are applied for augmentation. McKenna et al. compared the survival rates of both types of alveolar augmentation and reported an implant survival rate of 88.8% for implants inserted into iliac bone grafts after 120 months, which was significantly lower than the 95.2% survival rate of implants placed in intraorally harvested bone grafts. These authors mentioned the frequent donor site complications associated with iliac crest bone grafts [28].

### Limitations of the study

Studies analysing patient-related factors generally have to address the subjectivity of, e.g., wound evaluation by clinicians and interindividual differences in the pain perception of patients, which might have influenced the results of the current study. Compared with other donor sites, tibia bone grafts are rare grafts used in maxillofacial surgery, and the number of cases is limited. This has to be considered, especially when choosing appropriate statistical evaluation methods. Firth’s bias-reduced logistic regression is a method for small sample size analysis [16]. Owing to the small number of cases, the statistical power is low, but we can still show a tendency toward related risks and complication rates at the donor and recipient sites, which can be helpful for future clinical decision-making. Additionally, the data can contribute to future meta-analyses. The retrospective nature of the study led to the disadvantage of a non-standardized follow-up protocol, and the pre- and postsurgical care was based on the patient and wound demands. In addition, the exact defect size was not standardized for alveolar bone and nasal defects and this might have influenced the results at the recipient site. Nevertheless, all alveolar bone defects had in common, that they were combinations of vertical and horizontal bone deficiencies with a defect size between 2 and 4 cm.

### General considerations

Numerous criteria influence the decision about the best suitable tissue source, such as the required type and amount of tissue, the experience of the surgeon and, of course, the patient´s will. In addition, donor site morbidity and possible complications at the donor and recipient sites are other important factors that must be considered [27]. For extensive osseous reconstructions of the alveolar bone, e.g., due to atrophy before dental implant insertion, the iliac crest is currently one of the main sources for bone grafts. Bone harvesting from the iliac crest always takes place under general anaesthesia and is usually an inpatient operation. However, focusing on donor site morbidity and reducing surgical effort regarding the duration of surgery, the need for general anaesthesia and postoperative care the proximal tibia represents a promising alternative source for bone harvesting [6, 11, 22, 27, 31]. Alveolar augmentation due to alveolar atrophy after loss of dentition or alveolar cleft osteoplasty are typical indications for tibia bone grafts [9, 17, 29, 34]. The application of cortical tibia bone grafts for nasal augmentation represents a further clinical application, whereas most of the grafts are used for orthopedic reconstructions [14, 31]. A main argument in favour of harvesting the bone graft from the proximal tibia is the comparative lack of possible intra- and postsurgical complications compared with iliac bone grafts [6, 18]. Nevertheless, harvesting bone grafts from the proximal tibia is contraindicated in patients with open physes [31].

## Conclusion

The proximal tibia is a relevant donor site for bone grafts applied for alveolar augmentation and augmentative rhinoplasty because of the low donor and recipient site morbidity. Patient- and methodical-related factors were analysed for their influence on the complication rate, and only the season and form of anaesthesia while harvesting were found to have a significant impact. There was a tendency toward lower complication rates for augmentative rhinoplasty than for bone grafting for alveolar augmentation.

## Data Availability

No datasets were generated or analysed during the current study.
